# Gene Expression Responses to Sequential Nutrient Deficiency Stresses in Soybean

**DOI:** 10.3390/ijms22031252

**Published:** 2021-01-27

**Authors:** Jamie A. O’Rourke, Michelle A. Graham

**Affiliations:** Corn Insects and Crop Genetics Research Unit, USDA—Agricultural Research Service, Ames, IA 50010, USA; Michelle.Graham@usda.gov

**Keywords:** glycine max, IDC, iron stress, phosphate stress, abiotic stress, transcriptomics

## Abstract

Throughout the growing season, crops experience a multitude of short periods of various abiotic stresses. These stress events have long-term impacts on plant performance and yield. It is imperative to improve our understanding of the genes and biological processes underlying plant stress tolerance to mitigate end of season yield loss. The majority of studies examining transcriptional changes induced by stress focus on single stress events. Few studies have been performed in model or crop species to examine transcriptional responses of plants exposed to repeated or sequential stress exposure, which better reflect field conditions. In this study, we examine the transcriptional profile of soybean plants exposed to iron deficiency stress followed by phosphate deficiency stress (-Fe-P_i_). Comparing this response to previous studies, we identified a core suite of genes conserved across all repeated stress exposures (-Fe-P_i_, -Fe-Fe, -P_i_-P_i_). Additionally, we determined transcriptional response to sequential stress exposure (-Fe-P_i_) involves genes usually associated with reproduction, not stress responses. These findings highlight the plasticity of the plant transcriptome and the complexity of unraveling stress response pathways.

## 1. Introduction

Constantly changing environmental conditions expose field grown crops to a variety of stresses simultaneously, repeatedly, and sequentially. Because of this, plants have evolved specific mechanisms to detect and respond to environmental changes while conserving resources for proper growth and development [1]. Crop species were selected for traits associated with yield; however, in selecting for yield, other traits were likely impacted including stress tolerance. Understanding the molecular mechanisms underlying stress responses is the first step towards preserving crop yield under suboptimal growth conditions through either traditional breeding or engineering.

An abundance of studies have been conducted examining gene expression responses to single stress events. However, recent research has revealed that over 60% of gene expression responses to combined stress treatments were different than expected from single stress treatments [2]. For this reason, transcriptome studies have shifted to examine transcriptional changes under a combination of stresses. These types of studies will assist in identifying common responses shared by multiple stresses. Studies have revealed that *Arabidopsis* can very quickly alter its transcriptome when exposed to a second stress event [3]. Further, the transcriptional responses measured in sequential stress studies have revealed that initial stress exposure may alter a plants’ transcriptomic response to a second, or repeated, stress event, improving long-term plant fitness [4].

Iron (Fe) is one of fourteen essential micronutrients required for proper plant growth and development [5]. It is a key component to basic physiological processes including photosynthesis and electron transport. While abundant in soils, environmental conditions such as soil pH, composition, and oxygen availability often render it unavailable for use by plants [5]. This is especially problematic in the upper Midwestern United States where over 90% of U.S. soybeans are grown in calcareous soils. Soybeans grown under iron limited conditions exhibit interveinal yellowing of leaf tissue and reduced yield at the end of the growing season, with annual yield losses estimated to exceed USD 120 million [6,7,8].

Phosphorus (P), in its orthophosphate form (P_i_), is an essential macronutrient for all organisms. Like iron, it is plentiful in soils; however, little is available for plant use due to high fixation and slow diffusion [9]. Thus, despite its abundance, phosphorus is the most rate-limiting macronutrient in agricultural production [9]. Due to these factors, almost 30% of cropland around the world is P_i_ deficient [10]. To overcome this limitation and preserve yield, phosphorus is applied as a fertilizer. However, studies have shown plants only utilize 15–30% of applied fertilizer, with the rest lost to run-off, which contributes to the eutrophication of local and downstream water systems [11]. Further, phosphate fertilizer is derived from mined rock phosphate, a nonrenewable resource. Recent estimates suggest domestic P_i_ reserves will be depleted within the next 25–40 years, and importing P_i_ will become prohibitively more expensive [12,13].

The majority of studies elucidating the Fe and P_i_ uptake and homeostasis pathways have been performed in *Arabidopsis* and other model species [9,14,15,16,17,18,19,20,21,22]. However, the translation of these findings from model species to soybean and other crops has lagged behind and is often not straightforward [23,24]. For example, Peiffer et al. [7] fine mapped the major iron deficiency chlorosis quantitative trait locus (QTL) on soybean chromosome three and identified two candidate basic helix-loop-helix (bHLH) transcription factors with homology to *AtbHLH38* in *Arabidopsis*. AtbHLH38 interacts with AtFIT to regulate iron uptake [25]. However, silencing the two candidate genes in soybean had no phenotypic affect [26]. Recently, we combined a genome-wide association study with RNA-seq which split the QTL on soybean chromosome three into four distinct linkage blocks, each containing candidate genes explaining the breadth of the soybean iron stress response [26], and highlighting the complexity of quantitative traits in crop species.

Previous soybean studies provide an excellent foundation for understanding the genetic and molecular underpinnings of soybeans’ nutrient deficiency stress response and highlight the need to conduct these studies within crop species [26,27,28,29,30,31,32,33]. However, there is a shortage of studies examining the transcriptomic responses of soybean to sequential abiotic stresses, conditions faced regularly by crops. A previous study by our group examined the transcriptional response of soybean to single and repeated nutrient deficiencies (iron or phosphate), confirming that transcriptional responses to a repeated stress were not identical to the initial stress responses [34]. In the current study, we examine the transcriptomic responses of soybean exposed to -Fe stress followed by -P_i_ stress. Samples were grown, collected and analyzed simultaneously with those in our previous study, allowing for side by side comparisons. Specifically, we investigate whether transcriptomic responses to a micronutrient deficiency (Fe) followed by a macronutrient deficiency (P_i_) would be the same or different than repeated exposures of either stress. While examining the transcriptomic response of soybean to -Fe followed by -P_i_ only considers a single nutrient deficiency induced sequential stress response, we believe the broad findings of this study could be applied to or inform experimental designs for broader suites of studies in the future.

## 2. Results

### 2.1. Comparing Repeated Stress and Sequential Stress

**Figure 1:**
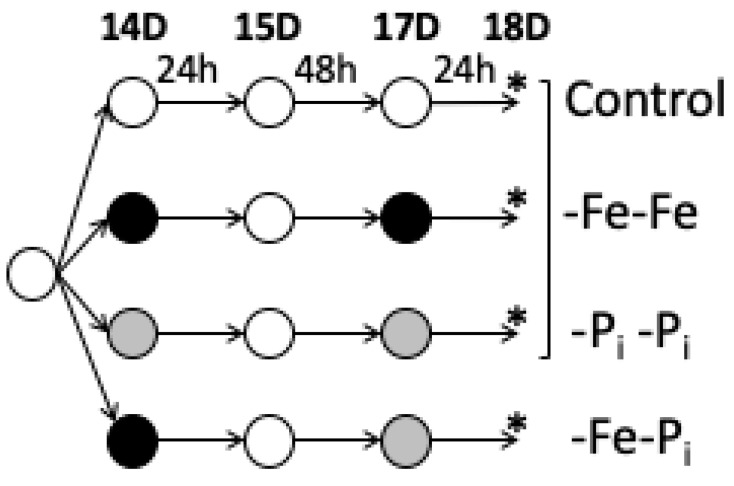
Experimental Design. Plants were sown in germination paper for seven days (D) and then transferred to full nutrient hydroponic solutions as described by [35]. The plant age (14D, 15D, 17D, and 18D post-germination) at corresponding experimental timepoints is provided at the top of the figure. At 14 days (14D), plants were moved to new nutrient solutions. Iron deficient (-Fe) nutrient solution, phosphate deficient (-P_i_) nutrient solution and full nutrient solution are indicated by black, grey and white circles, respectively. After 24 hours (h), all plants were moved to new full nutrient solutions for 48 h before a final move to new nutrient solutions for 24 h. At the end of the second 24 h stress exposure, leaf and root samples were harvested, denoted by an asterisk (*). For each tissue all 12 samples were grown, harvested, extracted, sequenced, and normalized together. Samples within bracket were analyzed and described in an earlier publication, [34]. However, to understand the biological importance of this treatment requires a comparison to previously published data.

Following the experimental design depicted in Figure 1 and the RNA isolation, sequencing, and analysis pipeline detailed in the materials and methods, statistical analyses identified 2975 and 10,612 DEGs in leaves and roots of -Fe-P_i_ stressed plants, respectively (Supplemental File 1: Tables S1 and S2). To identify similarities and differences between repeated stress and sequential stress exposures, we compared the -Fe-P_i_ differentially expressed genes (DEGs) from this study with the -Fe-Fe and -P_i_-P_i_ DEGs identified in our previous study [34], which was conducted simultaneously (Figure 1).

**Figure 2:**
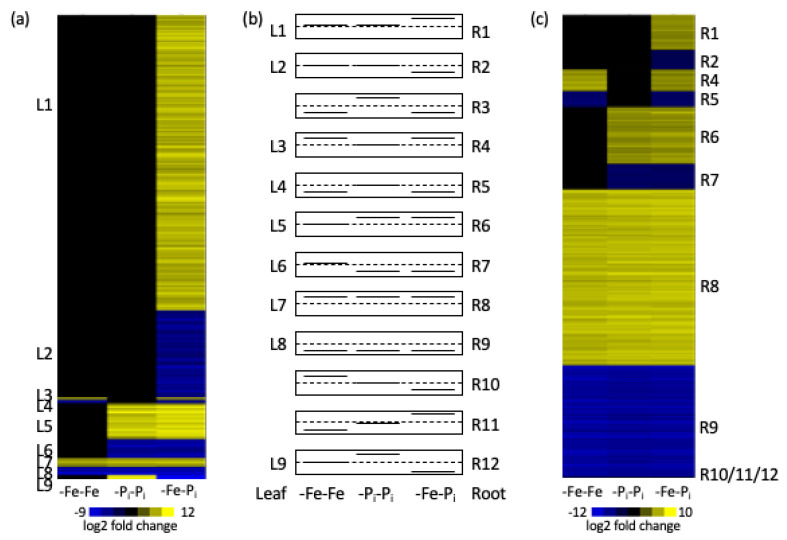
Distribution of statistically significant differentially expressed genes (DEGs) (**a**–**c**) in leaves (**a**) and roots (**c**) organized by possible expression profiles (**b**). Samples could either be downregulated (as indicated by blue in the heatmaps or by a solid line below the dashed line), not differentially expressed (black in heatmaps or solid line on the dashed line), or upregulated (yellow in heatmaps or solid line above the dashed line). Heatmaps present log2 fold changes compared to controls with minimum and maximum fold changes in each tissue provided with the expression scale. While 15 possible expression profiles were identified, only 12 profiles were identified in the data. Each cluster is assigned a unique designation. Samples from this experiment were exposed to iron deficiency followed by phosphate deficiency stress (-Fe-P_i_). These expression profiles were compared to a previous study examining repeated exposure to iron deficiency stress (-Fe-Fe) and repeated exposure to phosphate deficiency stress (-P_i_-P_i_) [34].

Between this study and our previous study [34] there were 15 possible gene expression profiles (Figure 2) depending on if a gene was significantly induced, repressed, or not differentially expressed in response to any of the three treatments (-Fe-Fe, -P_i_-P_i_ and -Fe-P_i_). Nine expression profiles could be found in both leaves and roots, three were specific to roots, and three were not expressed. As previously demonstrated, genes with similar expression patterns are often associated with the same biological processes [33,34]. Thus, we looked at the annotations of the genes for each of the 15 expression profiles. Statistical analyses found unique over-represented gene ontology (GO) terms for six of the nine expressed clusters in each tissue (Supplemental File 1: Table S3). GO analysis determined the DEGs in clusters L1 and L2 are associated with a variety of processes while R1 is associated with cell wall biosynthesis and R2 is associated with defense, specifically salicylic acid mediated signaling (Table 1 and Table 2, Supplemental File 1: Table S3). That genes with the same expression patterns, but in different tissues, are associated with such different biological processes serves to highlight the specialized roles different tissues play in abiotic stress responses.

**Figure 3:**
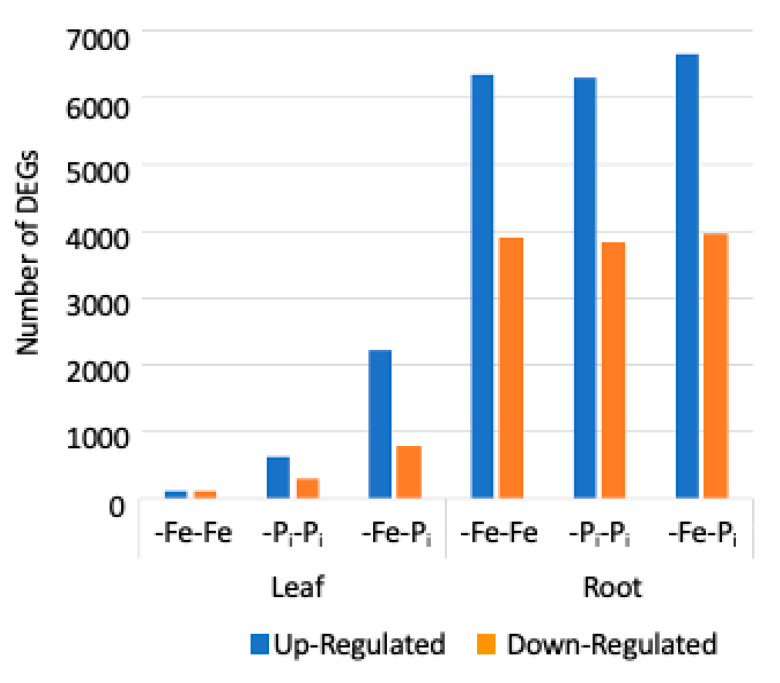
Distribution of DEGs in leaves and roots in this study (-Fe-P_i_) and in repeated stress exposure samples (iron stress, -Fe-Fe or phosphate stress, -P_i_-P_i_) from previous study [34]. The number of DEGs is significantly higher in leaves exposed to sequential stress (-Fe-P_i_) than in repeated stress samples while the number of DEGs in roots is consistent regardless of the stress exposure.

In roots, the majority of the DEGs (6608) are similarly differentially expressed in all three expression profiles. The GO processes associated with R8 and R9 include DNA replication, growth, photosynthesis, protein transport, and abiotic stress responses (Table 2, Supplementary File 1: Table S3). These are the hallmarks of the Clark genotype -Fe response [30]. Surprisingly in leaves, only 104 DEGs were differentially expressed in all three treatments; 52 upregulated and 52 downregulated (clusters L7 and L8). The genes in these two clusters represent the core soybean nutrient stress response, as supported by over-represented GO terms associated with stress responses (Table 1, Supplementary File 1: Table S3). It is noteworthy that in leaves the number of DEGs increases in response to sequential stress (Figure 3), while in roots the number of DEGs remains fairly constant in all three root stress profiles (Figure 3). In contrast, there are more DEGs in the root samples than in the leaf samples.

### 2.2. Identifying Genes Differentially Expressed in Both Leaves and Roots After -Fe-P_i_ Stress

**Figure 4:**
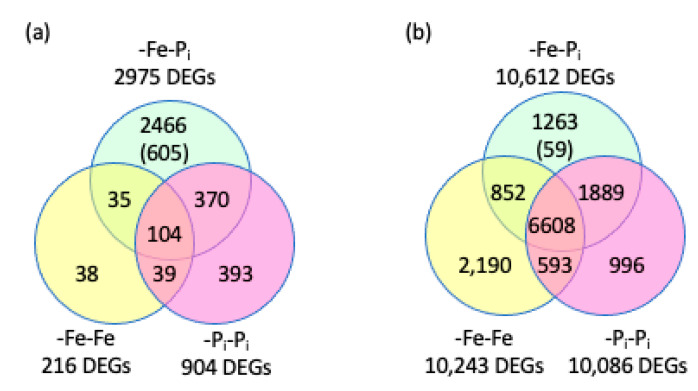
Differentially expressed genes (DEGs) identified in iron deficiency followed by phosphate deficiency (-Fe-P_i_) stressed soybean leaves (**a**) and roots (**b**) compared to previously published results of repeated iron deficiency stress (-Fe-Fe) or repeated phosphate deficiency stress (-P_i_-P_i_) leaves and roots [34]. Only genes within and overlapping with the -Fe-P_i_ circle are discussed throughout the manuscript. Numbers presented in parentheses represent the number of –Fe-P_i_ unique genes identified after altering parameters as described in Section 2.3 of the results.

**Figure 5:**
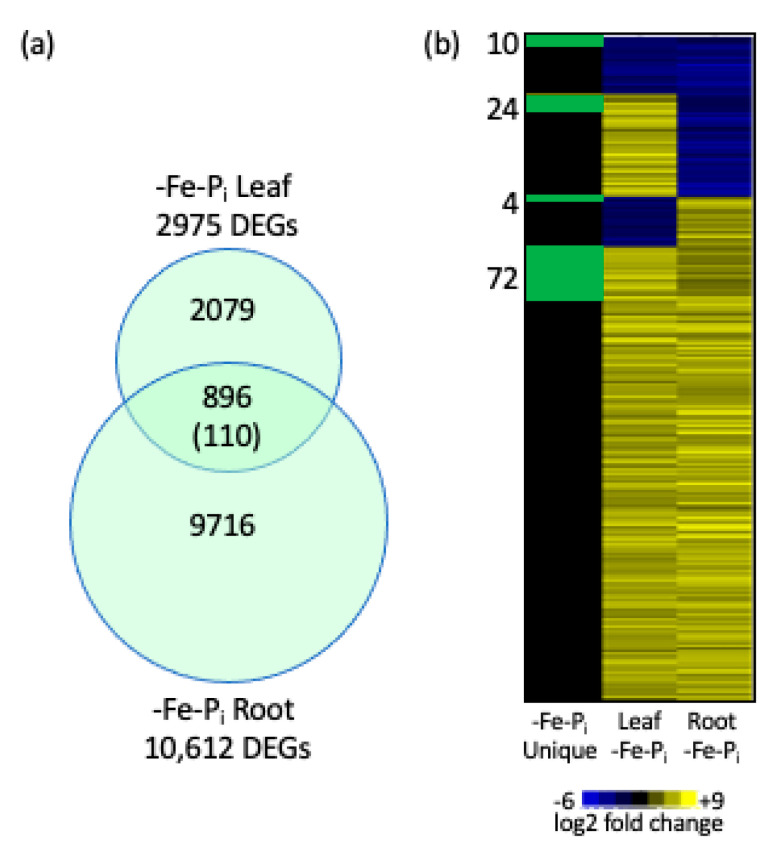
Gene expression conserved between tissues. There are 896 genes differentially expressed in both leaves and roots under -Fe-P_i_ growth conditions, with 110 genes unique to -Fe-P_i_ (**a**). Heatmap (using log2 expression) of the 896 DEGs common to -Fe-P_i_ tissues (**b**) yellow indicates gene expression is upregulated while blue indicates gene expression is downregulated compared to control samples. The distribution of the 110 genes unique to -Fe-P_i_ conditions is highlighted in green to the left of the heatmap and in column labeled -Fe-P_i_ unique.

In the leaves, the majority (82.8%, 2466) of DEGs were only significantly differentially expressed following -Fe-P_i_ sequential stress (Figure 4a). In the roots, only 1263 DEGs (11.9%) were specific to -Fe-P_i_ roots (Figure 4b). We also compared our results between tissues, identifying 896 DEGs common to -Fe-P_i_ leaves and roots with 110 of these specific to the -Fe-P_i_ treatment (Figure 5, Supplemental File 1: Table S4). This is interesting because previous studies have shown very little overlap in DEGs between these two tissues [29,33]. Gene annotation analysis identified 27 statistically significant GO terms associated with the 896 DEGs common to -Fe-P_i_ leaves and roots, all of which are associated with growth and/or defense. Additionally, GO analyses identified three statistically significant terms associated for the 110 -Fe-P_i_ specific DEGs, all of which are associated with secondary cell wall synthesis or programmed cell death (Supplemental File 1: Tables S5 and S6). Only a few of the 896 DEGs have annotations specifically associated with nutrient deficiency stress responses. These include *Glyma.02G003700* and *Glyma.10G183300*, homologs of *AtPHO1*, the major transporter of P_i_ into the root system [36]. PHO1 also plays an important role in mediating the leaf response to -P_i_ conditions [37]. Also conserved between roots and leaves are: *Glyma.06G052000*, a homolog of *AtIRT3*, a zinc and Fe transporter [38]; *Glyma.10G231600*, a homolog of *AtFRO4*, a ferric reductase regulated by FIT1 [39]; and *Glyma.16G168200* a homolog of *AtVIT*, a vacuolar Fe transporter responsible for mediating Fe homeostasis [40]. It is noteworthy that the majority of nutrient stress specific genes are associated with -Fe stress, suggesting that while -Fe stress responses have been well characterized in model species, genes associated with -P_i_ stress remain to be discovered. It is also noteworthy that the expression patterns of 690 of the 896 DEGs were conserved between tissues, with 612 upregulated in both tissues (Figure 5b, Supplemental File 1: Table S4). Annotations of the 690 genes indicate they are associated with plant growth and development. This is interesting since brief periods of nutrient deficiency stress and repeated nutrient stress exposure inhibits soybean growth and development processes [30,41,42].

### 2.3. Stringent Identification of Sequential Stress Specific Genes

This study was specifically designed to identify and analyze genes unique to the sequential stress exposure. As described in Section 4.3 of the materials and methods section, we recognized that the expression of some of these genes would be altered in either -Fe-Fe or -P_i_-P_i_, but not to a level exceeding statistical significance. To account for this, we relaxed the false discovery rate (FDR) (FDR < 0.25 -Fe-Fe and FDR < 0.25 -P_i_-P_i_) and removed the fold change cut-off requirements for the -Fe-Fe and -P_i_-P_i_ datasets. This allowed us to identify DEGs uniquely and significantly differentially expressed in the sequential stress response. This resulted in identification of 605 sequential stress specific genes in leaves and 59 in roots (Figure 4a,b, Supplemental File 1: Tables S1 and S2). Only a single gene (*Glyma.08G285300*) was differentially expressed in both leaves and roots. There are no over-represented GO terms affiliated with the 59 -Fe-P_i_ specific genes from roots. However, three GO terms (GO:0016126- sterol biosynthesis, GO:0010411- xyloglucan metabolism, and GO:0009620- response to fungus) representing 47 genes were overrepresented among the 605 DEGs specific to -Fe-P_i_ leaves. Since it is likely the initial -Fe stress primed the plant to quickly respond to repeated stresses, we searched the promoter regions of the -Fe-P_i_ specific genes for conserved motifs, representing likely transcription factor binding sites (TFBS). In the promoters of the 59 -Fe-P_i_ specific DEGs in roots, only three motifs were statistically over-represented (*p*-value <0.005); AT1G4765, MYB62, and MYB59 (Supplemental File 1: Table S7). In the promoter region of the 605 -Fe-P_i_ stress specific genes in leaves, there were 30 over-represented motifs including a number related to biotic and abiotic stress tolerance (Supplemental File 1: Table S8) including nine TFBS for bHLH TFs. These include a TFBS for BES1-INTERACTING MYC-LIKE 1 (BIM1), a bHLH TF known to be involved in brassinosteroid signaling [43], that was identified in the promoter of 315 of the 605 DEGs. Similarly, the TFBS for TCP8, which is important for systemic acquired resistance and directly promotes the expression of salicylic acid (SA) biosynthesis genes [44] was found in the promoter of 271 of the DEGs. In total, six homeodomain leucine zipper (HD-ZIP) TFBS were over-represented (EDT1, ATHB13, ATHB20, ATHB40, ATHB51, and ATHB53). These HD-ZIP TFBS were identified in the promoters of 373 DEGs. HD-ZIP TFs are known to regulate plant development and responses to biotic and abiotic stress in other species [45,46,47,48]. The identification of six HD-ZIP TFBS in the promoters of -Fe-P_i_ specific genes indicates these genes might play similar, and highly specialized, roles in the Clark sequential stress response.

## 3. Discussion

The gene expression analyses revealed five main findings. The first; there is a core suite of genes that is differentially expressed under all three stress conditions. Second, there is a subset of genes differentially expressed under -Fe-Fe, and -Fe-P_i_, but not -P_i_-P_i_ stressed plants. These genes represent a first stress signature. Third, some -P_i_ stress response genes are crucial to the -P_i_ response. These genes were differentially expressed under -P_i_-P_i_ and -Fe-P_i_. Fourth, there were more genes differentially expressed after sequential stress (-Fe-P_i_) than after repetitive stress (-Fe-Fe or -P_i_-P_i_) in both leaves and roots (Figure 4). Finally, there is a novel suite of genes differentially expressed under -Fe-P_i_ sequential stress that was not observed under either -Fe-Fe or -P_i_-P_i_. These represent enhanced stress responses generated by sequential stress application.

### 3.1. A Core Set of Genes Is Differentially Expressed in all Three Stress Profiles

First, we identified a suite of genes that is differentially expressed after all three stresses; representing the conserved core stress response. These 104 genes in leaves and 6608 genes in roots are the core responsive genes for nutritional deficiency response in the Clark genotype. Because there are so few genes associated with the individual clusters in leaves, we used all 104 genes from clusters L7 and L8 to look for over-represented gene ontology (GO) terms. In leaves the 104 DEGs are associated with terms involved in general stress responses (GO:0009408 response to heat, GO:0000160 phosphorelay signal transduction, GO:0009736 cytokinin mediated signaling, GO:2000121 regulation superoxide radical removal, GO:0042542 response to hydrogen peroxide, and GO:0034052 positive regulation of plant hypersensitive response). However, the annotations assigned to the genes associated with these GO terms are either involved in cytokinin signaling or heatshock proteins (HSPs). Cytokinin plays important roles in nutrient stress responses. Recent evidence suggests it might be a primary preceptor of nutrient sensing [49]. Cytokinin signaling is required for a strong response to -P_i_ [50] and suppresses genes for iron uptake and homeostasis [51]. Similarly, cytokinins indirectly regulate E2F transcription factors [52,53], which in turn regulate DNA replication genes. Atwood et al. [30] found E2F TFBS significantly overrepresented among genes responding to silencing of the DNA replication gene GmRPA3c. Silencing of this gene enhanced iron stress responses while limiting growth. Further, in biotic stress experiments cytokinin has been shown to act as a priming agent to prepare plants for enhanced responses upon biotic stress induction [54]. It is possible that cytokinin is serving as both a priming and signaling factor in this experiment. Recent research has shown HSPs respond to multiple biotic and abiotic stresses interacting with signaling molecules and play a key role in stress signaling networks [55,56,57].

The 6608 DEGs conserved across all three experimental profiles in roots can be assigned to one of three clusters in Figure 2 (clusters R3, R8, and R9). The expression of all but one gene is conserved in all three experimental profiles. Over-represented GO terms associated with the genes upregulated in all three expression profiles (cluster R8) are associated with growth, DNA replication/methylation, and photosynthesis (Table 2 and Supplemental File 1: Table S3). Modifying the transcriptional profiles of growth and DNA replication/methylation processes is a hallmark of the Clark iron deficiency response [26,30]. This is particularly interesting as genes associated with DNA replication and methylation are largely repressed after single stress exposure, regardless of stress duration [26,29,33,34]. The identification of 12 GO terms (343 genes) associated with photosynthesis reflects the importance of iron and phosphorus in photosynthetic process and the importance of photosynthates in the roots as an energy source. Twenty-two of the 35 over-represented GO terms associated with the 2563 downregulated genes (cluster R9) are associated with defense or abiotic stress responses (Table 2 and Supplemental File 1: Table S3). These include multiple GO terms associated with hormone biosynthesis and signaling including ethylene (GO:0009873, GO:0009723, GO:0010105), abscisic acid (GO:0009738 and GO:0009737), and jasmonic acid (GO:0010583 and GO:0009867). This reaffirms the important role hormones play in responding to abiotic stresses and is consistent with the findings of Coolen et al. [3], who hypothesized that the different stresses were interconnected by gene networks regulated by phytohormones.

### 3.2. Genes Required for -P_i_ Responses

The second finding in this dataset is the identification of 369 and 1889 genes essential for the -P_i_ stress response in leaves and roots, respectively (Figure 2 clusters L5, L6, and L9 in leaves and clusters R6, R7, and R12 in roots). These genes are differentially expressed in leaves after -P_i_-P_i_ and -Fe-P_i_ (but not -Fe-Fe), indicating they are required under -P_i_ growth conditions. 227 of the 369 P_i_ stress response genes in leaves belong to cluster L5 and are involved in P_i_ specific processes including galactolipid biosynthesis (GO:0019375), response to P_i_ starvation (GO:0016036), and phosphate ion homeostasis (GO:0030643) (Supplemental File 1: Table S3). Galactolipid membrane remodeling is a well-documented response to P_i_ deficiency stress in a variety of species [58,59] and has also been associated with disease resistance in soybean [60]. The annotations of the 21 DEGs in Figure 2 cluster L9 are associated with receptor like proteins (RLPs), leucine rich repeats (LRRs), and metal transport (Supplemental File 1: Table S1). This profile is noteworthy since in *Arabidopsis*, RLPs and LRRs regulate development, are known to confer resistance to a number of biological pathogens, and have been implicated in a number of abiotic stress tolerances [61,62,63]. Altered developmental regulation and defense responses are two hallmarks of the iron deficiency stress response in soybean. Our previous study [34] and work by McCabe et al. [60,64] associated RLPs with resistance to soybean brown stem rot, a disease often mistaken as iron deficiency chlorosis (IDC) due to the similarity of leaf symptoms likely caused by the pathogen damaging the vascular system, which prevents nutrient transport, and results in nutrient stress in leaves. The 1889 genes in roots are associated with general abiotic stress responses including growth and development, DNA replication and methylation, and hormone biosynthesis. The general stress responses in roots is consistent with previous findings that suggest roots arrive at a new homeostatic normality faster than leaves [34].

### 3.3. First Stress Signature Genes

Third, we identified a suite of 852 genes in roots (Figure 2 clusters R4, R5, R10, R11) and 35 in leaves (Figure 2 clusters L3 and L4) whose expression patterns mirror those observed in -Fe-Fe (Supplemental Files 1: Tables S1 and S2). These genes represent the “first stress signature” and illustrate the long-term impact of an early stress event on later gene expression patterns. Previous work by our research group has demonstrated that brief periods of -Fe stress early in a plant life cycle has long-term implications on gene expression patterns in older plants [34,65]. First stress signatures have been identified by a number of studies investigating sequential stress exposures [1,3,4]. In leaves, there are no over-represented GO terms associated with the 35 DEGs, but annotations of the closest *Arabidopsis* homologs indicate a number of them are involved in response to stress, cell wall modifications, and hormone biosynthesis or signaling. All these processes are known to be important in conferring abiotic stress tolerance. The hormone biosynthesis genes in particular are interesting since Coolen et al. [3] found first stress signatures were often related to phytohormone responses. This finding reinforces the idea that phytohormones are global modulators of stress interactions. In roots, the 361 genes in cluster R5 are associated with seven over-represented GO terms (Table 2 and Supplemental File 1: Table S3), all of which are associated with general stress response processes. In both leaves and roots, the majority of the first stress signature genes are expressed similarly in both -Fe-Fe and -Fe-P_i_. The similar expression pattern observed in the first stress signature genes highlights an important caveat of stress priming. While exposure to an initial stress can expedite and induce a stronger response to a second stress, it is not due to more extreme expression profiles of the same genes (i.e., transcription is not upregulated). Rather, it appears the enhanced response is due to a combination of faster responses and an increase in the number of genes recruited to the response.

It is worth noting that among the 852 first stress signature genes in roots, only two genes (*Glyma.19G132500* and *Glyma.07G171600*) are obviously associated with specific iron processes. *Glyma.19G132500* is homologous to *Glyma.03G130400*, which is located in the historical IDC QTL on Gm03 and is one of the bHLH38 putative candidate genes identified by Peiffer et al. [7]. In *Arabidopsis*, AtbHLH038 interacts with FIT to modulate expression of iron uptake genes and regulate iron homeostasis [18]. *Glyma.07G171600* is homologous to *AtbHLH121*, an upstream regulator of IRT1, which dimerizes with FIT to drive transcription of IRT1 and FRO2 [66]. Given the lack of iron specific genes, we examined the transcription factor binding sites over-represented in the promoter regions of the 852 genes (Supplemental File 1: Table S9). This analysis found 241 genes contain a binding site for bHLH34, Iron Deficiency Tolerant 1, which is important in regulating iron homeostasis in *Arabidopsis* [16]. This indicates that initial -Fe-P_i_ responses have already acted and the gene expression we measured 24 h after stress induction is a downstream response. Additional statistically significant TFBS identified in the promoter regions of the 852 DEGs are TFBS associated with brassinosteroid (BZR2, BIM1), cytokinin and auxin (SPT), abscisic acid (ABF2), and jasmonic acid (MYC3) signaling (Supplemental File 1: Table S9). The remaining over-represented TFBS identified in the 852 genes differentially expressed in both -Fe-Fe and -Fe-P_i_ roots are associated with regulating the balance between growth and defense, and general abiotic stress tolerance. We hypothesize that the genes containing these TFBS represent the growth/ development and defense branches of the Clark soybean iron deficiency response.

### 3.4. Sequential Stress Induces More DEGs in Leaves than in Roots

In our previous study [34], we compared gene expression levels after a single round of stress and after repetitive stress exposures and found that in leaves plants exposed to repetitive stresses exhibited far fewer differentially expressed genes compared to single stressed plants, while in roots the number of differentially expressed genes was nearly unchanged. In this study, more genes are differentially expressed after sequential stresses (-Fe-P_i_) than after repetitive stresses (-Fe-Fe or -P_i_-P_i_) in both tissues, though the increase is more dramatic in leaf DEGs (Figure 4). Given the previous results, it was surprising that sequential stress exposure in this study would dramatically increase the number of leaf DEGs but not root DEGs (Figure 3). However, a 2016 study by Coolen et al. [3] compared the transcriptomic response of *Arabidopsis thaliana* subjected to sequential biotic and abiotic stress exposures. This study found that sequential stress exposures shifted the timing of the expected changes in gene expression patterns. We know from previous studies that the number of DEGs in leaves increases through time (30, 60 and 120 min) after -Fe stress exposure [33] and root differential gene expression precedes leaf responses. Thus, it is logical that a short term initial -Fe stress exposure could reduce the time required to induce changes in the transcriptional response upon a second (-P_i_) stress, resulting in an increase in DEGs in -Fe-P_i_ stressed leaves compared to -Fe-Fe or -P_i_-P_i_ stressed leaves. Additionally, the differences we observed may reflect the different functions of iron and phosphate in the plant. As shown by our previous work [34] soybean plants utilize the same networks to uptake and transport iron and phosphate in the roots. However, the functions these nutrients play in growth and development are completely different. While iron is a key component of photosynthesis and electron transport [5], phosphate is found in DNA, RNA and proteins and is involved in the regulation of numerous plant processes including energy metabolism, and respiration [67]. Therefore, the application of a second, different nutrient stress is likely to have a larger impact on gene expression in the leaves.

### 3.5. Genes Unique to Sequential Stress Response

We identified 605 genes in leaves and 59 genes in roots (Figure 4, Supplemental File 1: Tables S1 and S2) that were not differentially expressed after either repetitive stress, even with the modified parameters described in the materials and methods, but were differentially expressed after sequential stresses were applied. Among the 605 genes unique to -Fe-P_i_ leaves, GO analysis found the processes sterol biosynthesis (GO:0016126), xyloglucan metabolism (GO:0010411), response to fungus (GO:0009620) and cellular metabolism (GO:0044237) are all over-represented (Supplemental File 1: Table S10). Previous studies have shown that increased sterol may induce stress response pathways [68]. This may be due to the role of sterols in lipid membranes or a downstream effect of the increased lipid signaling cascades induced by various abiotic stresses [69,70]. The modification of lipid membranes in response to -P_i_ is a common response, but this suite of genes has not previously been associated with -P_i_ deficiency responses. Further, the genes associated with xyloglucan metabolism are all involved in increased cell wall metabolism, a common stress defense response. We hypothesize these 605 genes are differentially expressed because the initial -Fe stress “primed” the plant and with the onset of a new novel stress (-P_i_), the plant induced an enhanced response to mitigate deleterious effects.

To better understand the 605 DEGs, we searched their promoters for over-represented TFBS (Supplemental File 1: Table S8). The transcription factors binding to these TFBS may be important regulators of the observed responses. These analyses identified 30 over-represented TFBS motifs in the promoter region of 589 of the 605 differentially expressed genes with most of the 605 promoters encoding multiple motifs. On average, each motif was identified in the promoter region of 185 genes. The TFBS motif for BIM1 (MA0964.1) was identified in the promoter region of 315 unique genes, the most for any motif. The BIM1 TF is involved in brassinosteroid signaling, modulating plant growth and development, and is required for male fertility [71,72,73]. Three additional over-represented motifs for TFs associated with brassinosteroids BIM2, BIM3, and BEE2 were also identified using this approach. Combined, these four motifs were identified in the promoters of 360 genes, emphasizing the importance of brassinosteroids in sequential stress responses. Previous studies have found brassinosteroids are important regulators of growth and development and are critical to modulating abiotic stress responses [74,75]. Not surprisingly, since hormones interact with each other to regulate responses, over-represented TFBS associated with other hormones were also identified in promoters of the 605 genes. Two TFBS are associated with ethylene (MA0980.2 (RAP2-10), and MA0995.2 (ERF039)), two with ABA (MA0123.1 (abi4), MA1209.1 (ATHB20)), two with cytokinin (MA1215.1 (ATHB53) and MA1061.1 (SPT)), two with gibberellic acid (MA1036.1 (MYB111) and MA1329.2 (ZHD1)) and two with salicylic acid (MA1197.1 (CAMTA1) and MA1428.1 (TCP8)). Additional TFBS motifs are associated with stress and defense responses. In total, 19 of the 30 motifs are involved in hormone biosynthesis and/or stress responses. The TFs associated with the stress response TFBS are associated with processes such as altering cell wall properties to modulate disease susceptibility, conferring broad spectrum disease resistance, systemic acquired resistance, and pathogen-associated molecular pattern (PAMP) triggered immunity (Supplemental File 1: Table S8). Finally, TFBS MA1197.1 is associated with the CAMTA1 TF, which is a master regulator of salicylic acid mediated immunity and a major driver of systemic acquired resistance (SAR) [76]. SAR is an important component of the stress priming process, facilitating a heightened and faster transcriptional response to a second stress exposure [77]. This motif was only found in the promoter region of 82 genes, which are involved in a variety of biological processes. Examining the annotations associated with the 605 DEGs found many are affiliated with altered cell wall architecture, which is to be expected. Unexpectedly, many also appear to be involved in seed development/germination processes and pollen. We hypothesize that two stresses applied in a sequential manner recruit the activity of genes normally reserved for specialized functions to quickly restore the plant to homeostasis. Testing of this hypothesis is beyond the scope of this paper, but it is worthy of future investigations.

In roots, only 59 genes (Figure 4, Supplemental File 1: Table S2) were unique to -Fe-P_i_ stress, 56 of which are upregulated. Given so few genes, no GO categories were statistically significantly over-represented. However, promoter analysis identified three TFBS motifs over-represented in the promoter regions of the 59 genes (Supplemental File 1: Table S7); none of which were over-represented in the 605 -Fe-P_i_ specific genes in leaves. The over-represented TFBS include AT1G47655 (57 genes), MYB59 (34 genes), and MYB62 (27 genes). While AT1G47655 has no known function, MYB59 regulates calcium signaling during growth and stress [78,79,80] and MYB62 regulates the phosphate starvation response and GA biosynthesis [81]. Given the limited insights provided by GO and Cis-element over-representation (CLOVER) analyses, we also examined the annotations associated with each of the 59 DEGs. Similar to the leaf expression patterns, regulation and development of the cell wall is represented. The upregulation of genes associated with the cell wall likely reflects the important role of the cell wall in the processes of defense, growth, and development. While the importance of slowing growth and development under nutrient deficient conditions is well documented, modifying the structural integrity of the cell wall is an important defense mechanism induced by multiple biotic and abiotic stresses [82,83,84]. Furthermore, DEGs in the roots are genes directly involved in iron deficiency stress. *Glyma.03G130400* is homologous to the *Arabidopsis bHLH038*, and resides within the major IDC QTL on soybean chromosome 3. In *Arabidopsis*, bHLH038 plays a major role in the -Fe stress response [18,25,85]. Also involved in the iron deficiency stress response is *Glyma.07G128000*, homologous to the *Arabidopsis* ACS7, which is involved in IDC induced ethylene biosynthesis [86]. In -Fe stressed *Arabidopsis*, ethylene production is upregulated and serves as an important signal to regulate -Fe responses [87,88,89]. Multiple genes involved in specific, known, stress responses are also unique to the root -Fe-P_i_ stress profile. These genes include *Glyma.13G101900*, *Glyma.01G230100*, and *Glyma.13G312700* whose homologs in *Arabidopsis* are involved in inducing SA mediated disease resistance [90,91], abiotic stress adaptation and negative regulation of PAMP triggered immunity [92], respectively. There are also three genes whose *Arabidopsis* homologs are involved in cation stress; (*Glyma.12G175000*) modulating cation transport [93,94], (*Glyma.17G001800*) mediating cadmium tolerance [95], and (*Glyma.13G127100*) and preventing cadmium toxicity by sustaining the TCA cycle and glutathione synthesis [96]. Cadmium and iron cations utilize the same transporters [17] and genes associated with cadmium homeostasis are likely involved in the homeostasis of other cations, including Fe^2+^. Of the 59 genes unique to -Fe-P_i_ roots with informative annotations, 50 are associated with either cell wall development or known stress responses, especially hormone biosynthesis responses. The association of these processes with -Fe-P_i_ stressed roots highlights their importance in the soybean stress response profile.

In conclusion, conducting experiments investigating transcriptomic responses to sequential stress exposure and repeated stress exposure simultaneously facilitated direct comparison between our two experiments. Analyses of these datasets determined sequential stress exposure induces novel responses, not just enhanced expression of genes differentially expressed in response to either micronutrient or macronutrient repeated stress exposure. Primary stress responses can be inferred from the TFBS analyses, which suggests hormones are likely serving as important signaling agents, but these insights will have to be confirmed by future experiments. While this experiment and analyses are illuminating, they also highlight the complexity of nutrient deficiency responses in crop species and reinforce the necessity of these studies in crop species.

## 4. Materials and Methods

### 4.1. Growth Conditions

Seeds from the soybean genotype Clark (PI:548533) were started on germination paper for seven days then transferred to hydroponic solutions described by Chaney, Coulombe, Bell and Angle [35]. Cotyledons were removed on day of transfer. All plants were grown in full nutrient solutions for seven days. After seven days, half of the plants were moved to -Fe (50 µM Fe(NO_3_)_3_) and half were moved to new full nutrient solutions. After 24 h all plants were again moved to new full nutrient solutions for 48 h. After 48 h, the -Fe stressed plants were moved to -P_i_ (-Fe-P_i_) and the control plants were again moved to new full nutrient solutions. After 24 h in the new nutrient solutions, the fourth trifoliate and the entire root system of four biological replicates, each a single plant, was harvested and immediately frozen in liquid nitrogen.

Plants for this experiment were grown simultaneously, as a subset of a larger experiment, previously described by O’Rourke, McCabe and Graham [33]. In brief, O’Rourke, McCabe and Graham [33] generated samples for eight different treatments: early stress (-FeT1 and -PiT1), stress recovery (-FeT1Rec and -PiT1Rec), repeated stress (-FeT1T2 and -PiT1T2), late stress (-FeT2 and -PiT2), and nonstress controls collected at each timepoint. The layout of the entire experiment is depicted in Figure 1. The experiment, RNA isolation, and data analyses were conducted simultaneously, to allow direct comparison of results. For ease of interpretation, the repeated stress experiments from O’Rourke, McCabe and Graham [33] will now be referred to as -Fe-Fe (formerly -FeT1T2) and -Pi-Pi (formerly -PiT1T2).

### 4.2. RNA Isolation

RNA was extracted using Qiagen RNeasy kits (Qiagen, Valencia, CA). Contaminating DNA was removed using the Ambion TURBO DNA-free kit (Ambion, Austin, TX, USA). RNA was purified and concentrated using the RNeasy MinElute Cleanup kit (Qiagen, Germantown, MD, USA). Sample purity and quantity was measured using the nanodrop ND-1000 spectrophotometer (ThermoFisher Scientific, Waltham, MA, USA) and QiaCel (Qiagen, Germantown, MD, USA). RNA from three biological replicates was submitted to the Iowa State University DNA Facility for sequencing. All reads have been submitted to the National Center for Biotechnology Information (http://ww.ncbi.nih.gov/sra) under BioProject accessions PRJNA544698 and PRJNA662977.

### 4.3. RNA-Seq and Data Analysis

Library preparation was performed from 4 µg of total RNA and subsequent 100 bp single end sequencing was performed using the Illumina HiSeq2500 (Illumina, San Diego, CA, USA). Reads with quality scores greater than 20 and longer than 30 bases were mapped to the soybean genome (Glyma.Wm82.a2.v1, (Gmax2.0), https://phytozome.jgi.doe.gov/pz/portal.html#!info?alias=Org_Gmax) using Tophat2 (v2.1.1, [96]) with default parameters except a maximum intron length of 10,000 base pairs. The program samtools (v1.3.1, [97]) was used to retain uniquely mapping reads. Sample data were imported into R-studio (v0.98.945, [98]) for further analysis. Leaf and root samples were normalized independently using DESeq (v1.14.0, [99]). The graphics program ggplot2 (v0.9.3.1, [100]) was used to visualize expression between replicates to ensure consistency. This analysis determined the expression profile of a single control leaf sample was statistically different from the biological replicates. This sample was removed from the analyses and the data renormalized. Using the renormalized data, edgeR [101] analyses identified DEGs. Samples from the original study (O’Rourke, et al., 2020), were extracted, sequenced and normalized with samples from the current study, facilitating direct comparisons. Differential expression analyses compared plants exposed to nutrient stress to plants grown continuously in full nutrient conditions at the same timepoint. DEGs were considered significant if their fold change was >2, *p*-value was <0.05 and the FDR was <0.05. Gene expression profiles for all DEGs identified in leaves and roots are available in Supplemental File 1: Tables S1 and S2, respectively. To identify genes that were unique to -Fe-P_i_ sequential stresses we compared the list of DEGs identified in -Fe-P_i_ leaves and roots to leaf and root DEG lists from soybeans exposed to repeated -Fe stress and repeated -P_i_ stress as described previously [33]. To identify genes uniquely differentially expressed in -Fe-P_i,_, the FDR for -Fe-Fe and -P_i_-P_i_ genes was raised to FDR < 0.25 and the log fold change >1 ratio was removed.

### 4.4. Gene Annotation

Annotations for each of the DEGs were assigned using the annotation tool on SoyBase (www.soybase.org/genomeannotation/). Primary proteins of the *Glycine max* v2 genome were compared to all available *Arabidopsis thaliana* proteins (www.TAIR.org, version 10) using BLASTP (E < 10^−6^). The best hit is reported. Over-represented gene ontology (GO) terms associated with different gene lists were identified using the GO term enrichment tool on SoyBase (https://www.soybase.org/goslimgraphic_v2/dashboard.php). This tool assigns gene ontology terms to each soybean gene using the gene ontology (GO) of the best *Arabidopsis thaliana* homolog as identified by BLASTP (E < 10^−6^) then uses a Fisher’s exact test [102] with a Bonferroni correction [103] as described in Morales et al. [104]. Overrepresented GO terms were used to assign biological function and classification to heatmap clusters. Transcription factors (TFs) were identified using the SoyDB transcription factor database published by Wang, et al. [105]. Overrepresented TFs were identified using the same methodology for over-represented GO terms.

### 4.5. Identification of Over-Represented Transcription Factor Binding Sites

To identify over-represented (*p*-value cutoff of 0.005) transcription factor binding sites (TFBS) within the promoters of gene lists of interest relative to all promoters in the current genome assembly, we used CLOVER [106] and the JASPAR (v8) Transcription Factor database [107]. Custom perl scripts were used to extract 500 bases of promoter sequence from genes of interest and for all genes in the genome (Glyma.Wm82.a2.v1). Promoters less than 500 bases or containing gaps or ambiguous bases were removed from the analysis.

## Figures and Tables

**Table 1:** Top five GO terms associated with leaf expression profiles corresponding to Figure 2.

Cluster	Pattern	# DEGs	Corrected *p*-Value	GO ID	GO Description
L1	0,0,+	1908	1.69 × 10^−8^	GO:0009733	Response to auxin stimulus
			2.25 × 10^−7^	GO:0043481	Anthocyanin accumulation, UV response
			9.91 × 10^−5^	GO:0010817	Regulation of hormone levels
			1.26 × 10^−4^	GO:0009611	Response to wounding
			5.40 × 10^−4^	GO:0055114	Oxidation-reduction process
L2	0,0,−	558	0.016700	GO:0042754	Negative regulation of circadian rhythm
L3	+,0,+	15			No stat sig GO
L4	−,0,−	20			No stat sig GO
L5	0,+,+	227	4.06 × 10^−4^	GO:0016036	Response to phosphate starvation
			4.83 × 10^−3^	GO:0019375	Galactolipid biosynthetic process
			4.05 × 10^−3^	GO:0030643	Cellular phosphate ion homeostasis
L6	0,−,−	121	3.93 × 10^−5^	GO:0000103	Sulfate assimilation
			0.017285	GO:0006792	Regulation of sulfur utilization
			0.028727	GO:0010438	Cellular response to sulfur starvation
			0.028727	GO:0019419	Sulfate reduction
L7	+,+,+	52	0.002435	GO:0000160	Phosphorelay signal transduction
			0.003631	GO:2000121	Regulating superoxide radical removal
			0.012052	GO:0034052	Positive regulation of plant hypersensitive response
L8	−,−,−	52	2.58 × 10^−9^	GO:0009408	Response to heat
			0.001374	GO:0042542	Response to hydrogen peroxide
			0.004119	GO:0009644	Response to high light intensity
			0.016569	GO:0006110	Regulation of glycolysis
			0.039347	GO:0006979	Response to oxidative stress
L9	0,+,−	21			No stat sig GO

Cluster corresponds to list designation in Figure 2. Pattern reflects the gene expression pattern in -Fe-Fe, -P_i_-P_i_, and -Fe-P_i_ samples: 0 indicates no significant change in expression, + indicates upregulated expression, − indicates downregulated expression. # DEGs indicates the number of differentially expressed genes assigned to each cluster. Corrected *p*-value is calculated using a Fisher’s exact test with a Bonferroni correction. Short horizontal lines separate information for multiple GO terms assigned to the same cluster.

**Table 2:** Top five gene ontology (GO) terms associated with root expression profiles corresponding to Figure 2.

Cluster	Pattern	# DEGs	Corrected *p*-Value	GO ID	GO Description
R1	0,0,+	813	2.96 × 10^−15^	GO:0009834	Secondary cell wall biogenesis
			1.97 × 10^−7^	GO:0010413	Glucuronoxylan metabolism
			2.19 × 10^−7^	GO:0045492	Xylan biosynthetic process
			4.33 × 10^−6^	GO:0044036	Cell wall macromolecule metabolism
			0.000167	GO:0046274	Lignin catabolic process
R2	0,0,−	450	0.002135	GO:0009863	Salicylic acid mediated signaling
			0.002228	GO:0002679	Respiratory burst in defense
R3	−,+,−	1			No stat sig GO
R4	+,0,+	487			No stat Sig GO
R5	−,0,−	361	5.30 × 10^−5^	GO:0009715	Chalcone biosynthetic process
			9.67 × 10^−5^	GO:0009629	Response to gravity
			5.91 × 10^−4^	GO:0010224	Response to UV-B
			0.011275	GO:0006979	Response to oxidative stress
			0.016313	GO:0031540	Regulation of anthocyanin biosynthesis
R6	0,+,+	1305	4.32 × 10^−12^	GO:0009832	Plant-type cell wall biogenesis
			1.13 × 10^−9^	GO:0007018	Microtubule-based movement
			2.39 × 10^−9^	GO:0030243	Cellulose metabolic process
			4.26 × 10^−8^	GO:0016126	Sterol biosynthetic process
			1.95 × 10^−7^	GO:0010075	Regulation of meristem growth
R7	0,−,−	583			No stat sig GO
R8	+,+,+	4044	6.25 × 10^−40^	GO:0000911	Cytokinesis by cell plate formation
			1.28 × 10^−32^	GO:0008283	Cell proliferation
			3.40 × 10^−32^	GO:0010075	Regulation of meristem growth
			1.40 × 10^−29^	GO:0006275	Regulation of DNA replication
			1.28 × 10^−26^	GO:0010389	Regulation of G2/M transition of mitosis
R9	−,−,−	2563	2.02 × 10^−25^	GO:0006606	Protein import into nucleus
			2.32 × 10^−18^	GO:0006626	Protein targeting to mitochondrion
			2.46 × 10^−18^	GO:0001510	RNA methylation
			4.35 × 10^−15^	GO:0034976	Response to ER stress
			3.01 × 10^−12^	GO:0009220	Pyrimidine ribonucleotide biosynthesis
R10	+,0,−	1			No stat sig GO
R11	−,0,+	3			No stat sig GO
R12	0,+,−	1			No stat sig GO

Cluster corresponds to list designation in Figure 2. Pattern reflects the gene expression pattern in -Fe-Fe, -P_i_-P_i_, and -Fe-P_i_ samples: 0 indicates no significant change in expression, + indicates upregulated expression, - indicates downregulated expression. # DEGs indicates the number of differentially expressed genes assigned to each cluster. Corrected *p*-value is calculated using a Fisher’s exact test with a Bonferroni correction. Short horizontal lines separate information for multiple GO terms assigned to the same cluster.

## Data Availability

Datasets associated with this study can be found in the short read archive (SRA) database (http://ww.ncbi.nih.gov/sra) under BioProject accessions PRJNA544698 and PRJNA662977.

## Supplementary Materials

Supplementary materials can be found at https://www.mdpi.com/1422-0067/22/3/1252/s1; Supplemental file 1: Table titles. Table S1. All DEGs in -Fe-Pi leaves. Table S2. All DEGs in -Fe-Pi roots. Table S3. Over-represented GO terms associated with expression profiles depicted in Figure 2. Table S4. The number of -Fe-Pi DEGs conserved between root and leaf tissues was 896. One hundred and ten DEGs specific to -Fe-Pi treatment are denoted. Table S5. GO terms associated with the 896 DEGs conserved between root and leaf tissues. Table S6. GO terms associated with the 110 DEGs both conserved between tissues and specific to the -Fe-Pi treatment. Table S7. Over-represented transcription factor binding sites identified using CLOVER in 59 -Fe-Pi specific DEGs in roots. Table S8. Over-represented transcription factor binding sites identified using CLOVER in 605 -Fe-Pi specific DEGs in leaves. Table S9. Over-represented transcription factor binding sites identified using CLOVER in 852 “First Stress Signature” genes in roots. Table S10. Over-represented GO terms identified among 605 DEGs unique to -Fe-Pi leaves.

## Abbreviations

DEGs	Differentially expressed genes
FDR	False discovery rate
-Fe	Iron deficiency stress
-Fe-Fe	Iron deficiency stress (repeated stress)
-Fe-P_i_	Iron deficiency stress followed by phosphate deficiency stress (sequential stress)
GO	Gene ontology
h	Hours
IDC	Iron Deficiency Chlorosis
*p*	Probability
-P_i_	Phosphate deficiency stress
-P_i_-P_i_	Phosphate deficiency stress (repeated stress)
TF	Transcription factor
TFBS	Transcription factor binding site
TFF	Transcription factor family
